# Supplementary material for: Mid-cell migration of the chromosomal terminus is coupled to origin segregation in Escherichia coli
Source: Nat Commun. 2023 Nov 18;14:7489. doi: 10.1038/s41467-023-43351-7 (PMC10657355; doi:10.1038/s41467-023-43351-7)
Supplement: Supplementary file 1 — Supplementary Information [file 41467_2023_43351_MOESM1_ESM.pdf]

Supplementary Information

Supplementary Figures

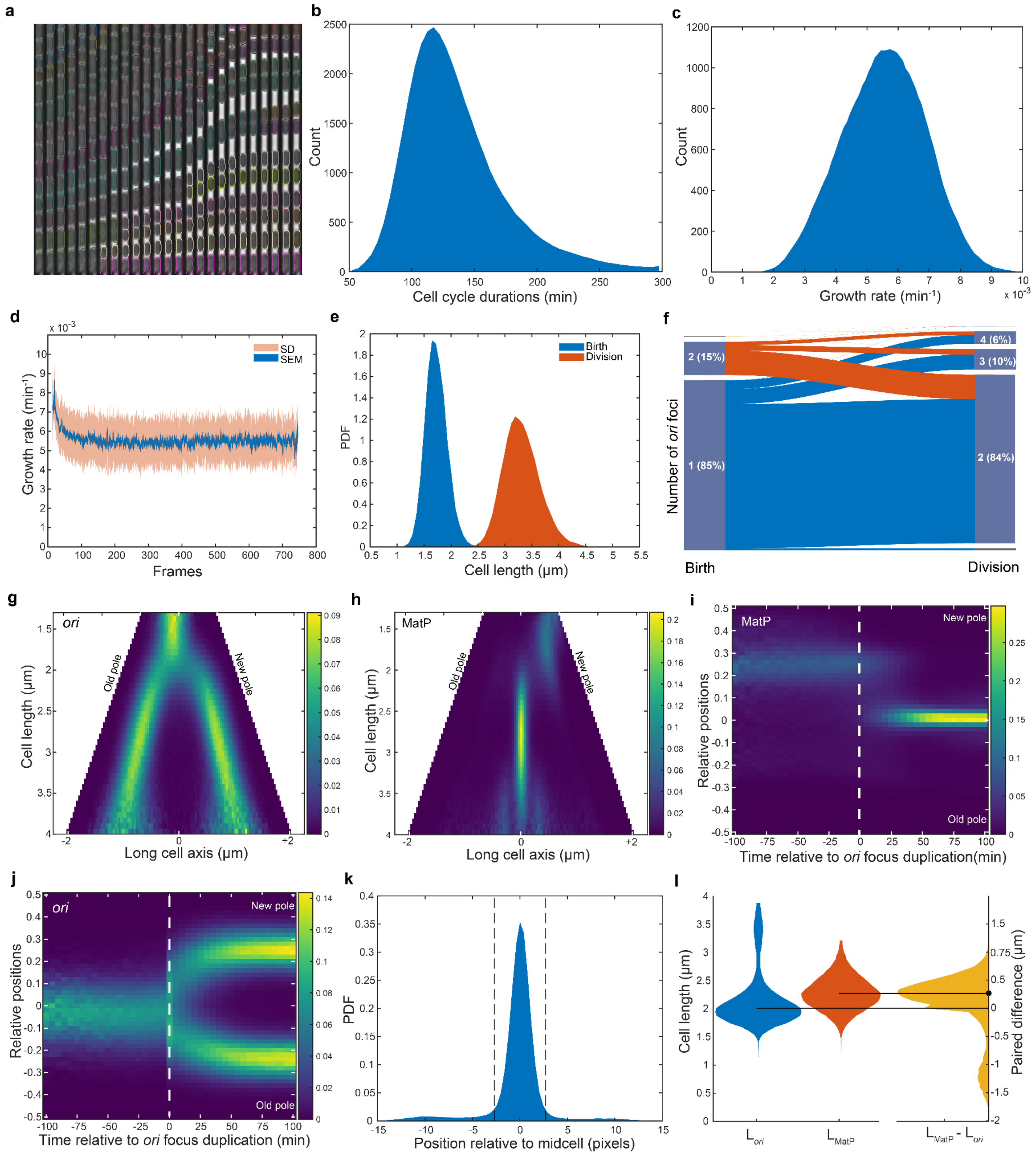

Supplementary Figure 1. **Further analysis of the data in Figure 1 (strain IS 130). a** An example of segmentation and tracking of cells in a growth channel. **b** Histogram of cell cycle durations (mean ± sd = 132.8 ± 39.1 min) for the dataset used in Figure 1 and S1 (n=38066 cell cycles) **c** Histogram of growth rates of individual cell cycles ((5.5 ± 1.3) x 10^{-3} min^{-1}). **d** Mean growth rate as function of time (frames) in the device showing stable growth conditions throughout the imaging. **e** Probability density function function (PDF) of birth (1.71 ± 0.2 μm) and division lengths (3.31 ± 0.33 μm). **f** Flow diagram showing the number of *ori* foci at birth and division. **g** Demograph of *ori* foci positions along the long axis of cells binned according to cell lengths. **h** Demograph of MatP foci positions along the long axis of cells binned according to cell lengths. **i** Kymograph of MatP foci positions relative to the frame of *ori* foci duplication. **j** Kymograph of *ori* foci positions relative to the frame of *ori* foci duplication. **k** Probability density function of MatP foci positions in cells between 2.5 μm and 3 μm. **l** Distribution of cell lengths at *ori* duplication and MatP centralisation along with their paired difference as in Figure 1c. The smaller peak for L_{ori} near 3.5 μm shows that a portion of cells duplicates *ori* twice in a cell cycle (see S1F). The colour scale in **i** and **j** is as in Figure 1. The values in the colour scale for the demographs **g** and **h** represent the frequency of occurrence of foci positions normalised to the number of cells at each cell length. The growth rate in **c** and **d** is calculated using an exponential fit to the cell area for each cell cycle. Source data are provided as a source data file.

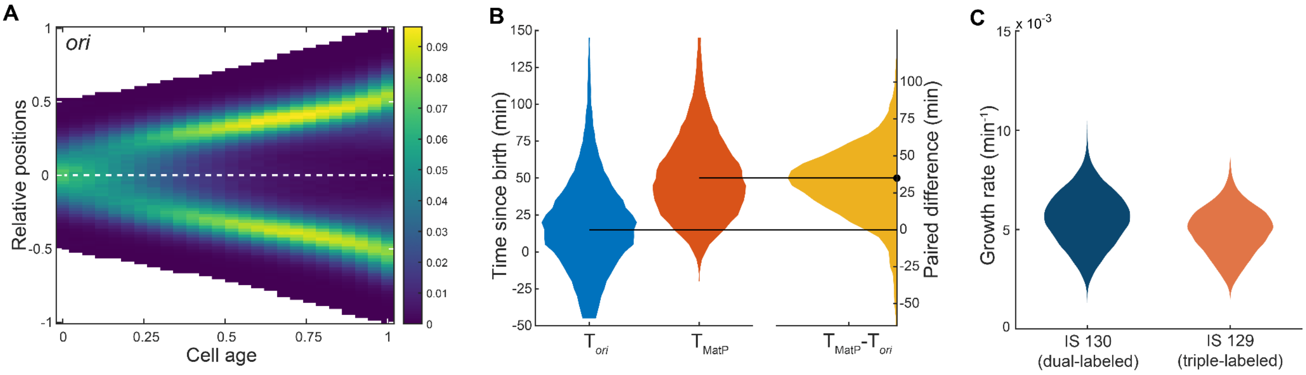

Supplementary Figure 2. **Further analysis of the data in Figure 2. a** Average kymograph of *ori* foci positions along the long axis of the triple labelled strain (IS 129) from Figure 2 but with cells oriented randomly, i.e, not according to old-pole and new-pole **b** Distribution of the time of *ori* focus duplication, T_{ori} (mean ± sd = 19.1 ± 31.3 min) and MatP, T_{MatP} centralization (50.7 ± 28.0 min) along with the time difference between the two events (31.6 ± 25.2 min) as in Figure 1C **c** Growth rate distribution of the dual labelled strain IS 130 used in Figure 1 ((mean ± sd = (5.5 ± 1.3) x 10^{-3} min^{-1}) and triple labelled strain IS129 used in Figure 2 ((4.9 ± 1.2) x 10^{-3} min^{-1}). The mean doubling times of IS 130 and IS 129 are 132.8 ± 39.1 minutes and 142.7 ± 43.5 minutes. The triple labelled strain grows 11% more slowly. Growth rate is calculated using an exponential fit to the cell area for each cell cycle. The colour scale in **a** is as in Figure 1. Source data are provided as a source data file.

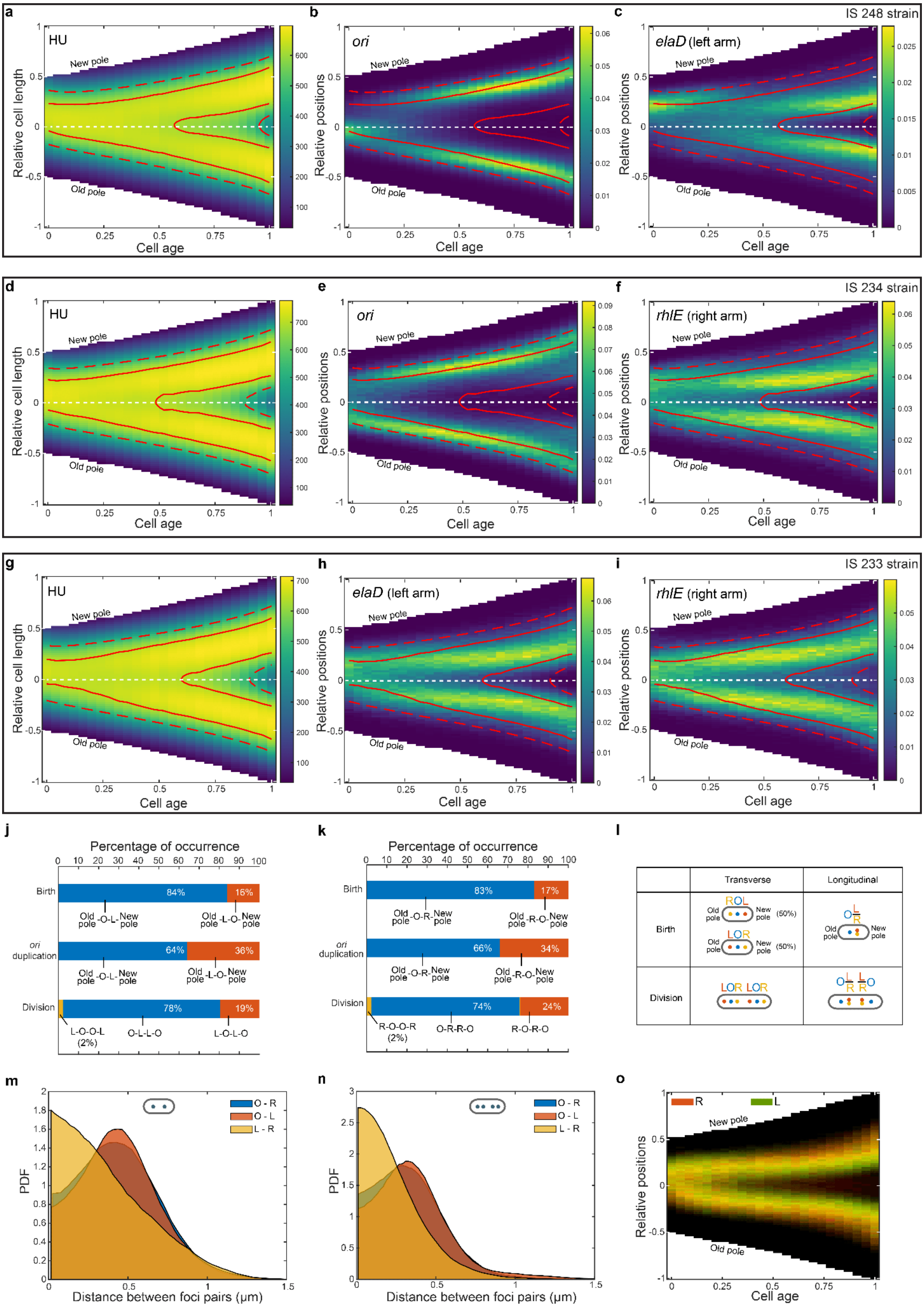

a
HU
New pole
Old pole
Relative cell length
Cell age
b
ori
Relative positions
c
IS 248 strain
elaD (left arm)
d
e
f
IS 234 strain
rhlE (right arm)
g
h
i
IS 233 strain
j
Percentage of occurrence
Birth
84%
16%
Old pole -O-L- New pole
Old pole -L-O- New pole
ori duplication
64%
36%
Division
78%
19%
L-O-O-L (2%)
O-L-L-O
L-O-L-O
k
83%
17%
Old pole -O-R- New pole
Old pole -R-O- New pole
66%
34%
74%
24%
R-O-O-R (2%)
O-R-R-O
R-O-R-O
l
Transverse
Longitudinal
Birth
ROL
LOR
(50%)
Division
m
PDF
O - R
O - L
L - R
Distance between foci pairs (μm)
n
o
R
L

Supplementary Figure 3. **The chromosome has a longitudinal organisation.** Analysis of strains in which either the left-arm *elaD* locus (IS248) (**a-c**), the right-arm *rhlE* locus (IS234) (**d-f**) or both (IS233) (**g-i**) are tagged. **a, d, g** Average kymograph of HU-mCherry signal along the long axis of the cell for each of the strains. The solid contour lines represent upper 50 percent, and the dashed lines represent upper 80 percent of the total HU-mCherry signal for the respective strain. **b, e** Average kymograph of foci positions of *ori* labelled with mTurquoise2-ParB$_{P1}$ in the *elaD* (**b**) and *rhlE* (**e**) tagged strains. The contour lines correspond to the respective HU-mCherry signal in each strain. **c, f** Average foci kymograph of foci positions of *elaD* (**c**) and *rhlE* (**f**) loci labelled with mVenus-ParB$_{pMT1}$. **h, i** Average kymograph of foci positions of *elaD* and *rhlE* loci labelled using mVenus-ParB$_{pMT1}$ and mTurquoise2-ParB$_{P1}$ respectively in the same strain. **j, k** Occurrence of different chromosome orientations in the *elaD* (**j**) and *rhlE* (**k**) labelled strains at birth, division and in the frame immediately prior to visible *ori* duplication. O: *ori*; L: *elaD*; R: *rhlE*. **l** Schematic representation of the expected chromosome orientations in the transverse and longitudinal models of chromosome organisation. **m** Distance between foci pairs (O-L, O-R, L-R) for cells with two foci in strains IS 234 (blue), IS 248 (red) and IS 233 (yellow). The mean ± 95% CI distances are 0.436 ± 0.006 µm, 0.446 ± 0.003 µm and 0.368 ± 0.004 µm respectively. **n** Distance between foci pairs (O-L, O-R, L-R) for cells with four foci in IS 234 (blue), IS 248 (red) and IS 233 (yellow). The mean ± 95% CI distances are 0.333 ± 0.002 µm, 0.362 ± 0.002 µm and 0.239 ± 0.001 µm respectively. **o** Kymograph showing the overlay of *elaD* (L) and *rhlE* (R) loci positions (**h, i)** from IS 233. Data for the *elaD* (IS 248)*, rhlE* (IS 234) and dual labelled (IS 233) strains are from n=31344, 6240 and 6420 cell cycles respectively. Source data are provided as a source data file.

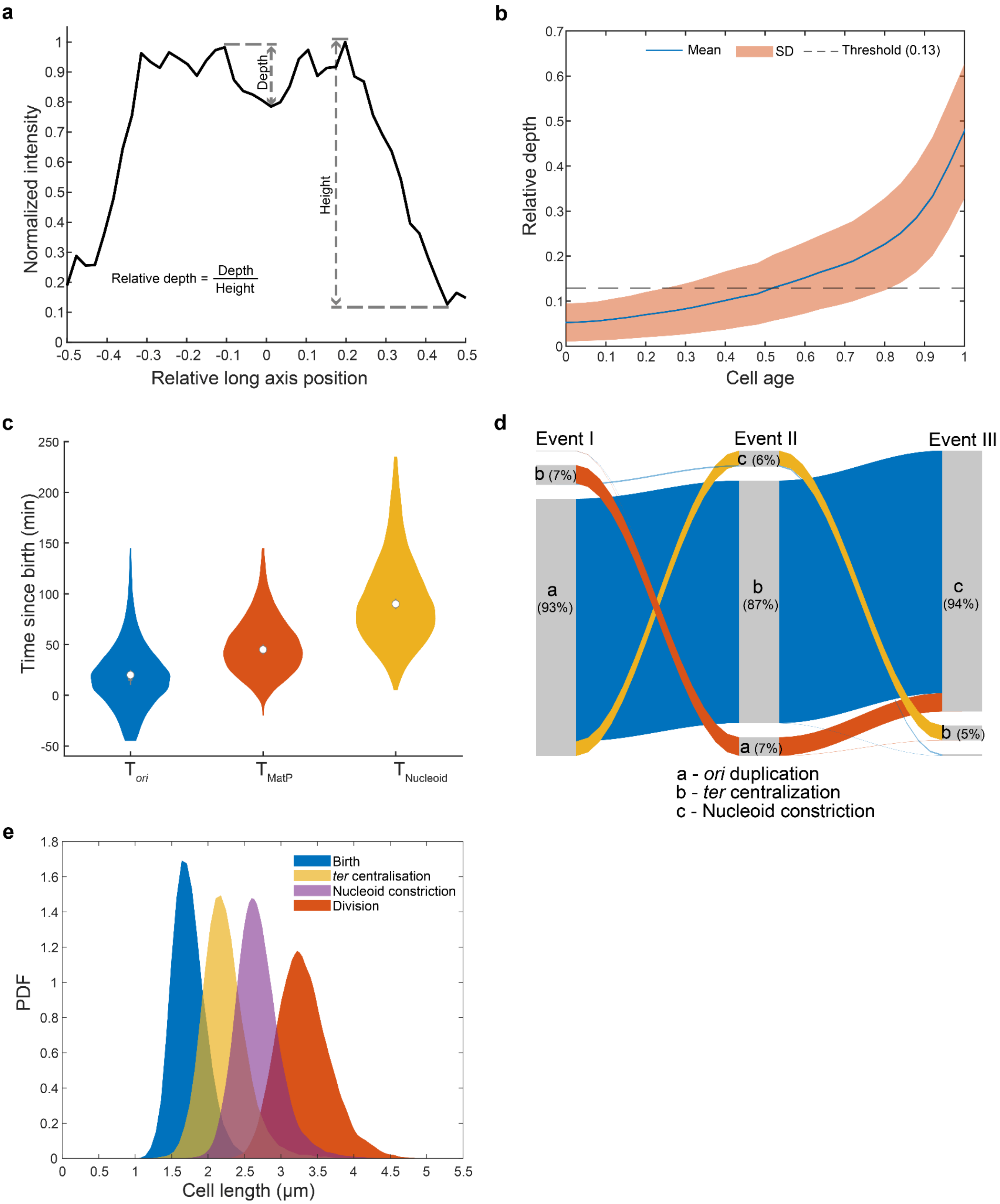

Supplementary Figure 4. **Further analysis of the data in Figure 3. a** Line profile from Figure 3a labelled with parameters used for determining nucleoid constriction. **b** Relative depth of HU-mCherry signal binned according to the cell age (24 bins). The threshold (0.13) for nucleoid constriction is defined as the value at the 95th percentile of the first bin based on the assumptions that no new born cell has a truly constricted nucleoid so that any dips observed are due to random fluctuations. The blue line represents the mean and the shaded region represents standard deviation **c** Distribution of time of *ori* focus duplication, T_{ori} (mean ± s.d = 20.0 ± 32.3 min) MatP centralization, T_{MatP} (49.7 ± 28.6 min) and stable nucleoid constriction, $T_{Nucleoid}$ (95.0 ± 44.3

min). The white dots indicate the mean. The time of stable nucleoid constriction corresponds to the earliest frame from which the nucleoid maintains a dip greater than the threshold. Paired differences are given in Supplementary Figure 2b and Figure 3b **d** Order of occurrence of events involving *ori* duplication, *ter* centralization and continuous nucleoid constriction. a, b and c represent the events *ori* duplication, *ter* centralization and nucleoid constriction respectively. The corresponding percentages indicate the proportion of cells in which a particular event was first (Event **I**), second (Event **II**) or third (Event **III**) to occur. **e** Probability density function (PDF) of cell lengths at which T_{MatP} (2.22 ± 0.30 μm) and $T_{Nucleoid}$ (2.68 ± 0.30 μm) occur in cell cycles along with their birth (1.73 ± 0.22 μm) and division lengths (3.32 ± 0.36 μm). Source data are provided as a source data file.

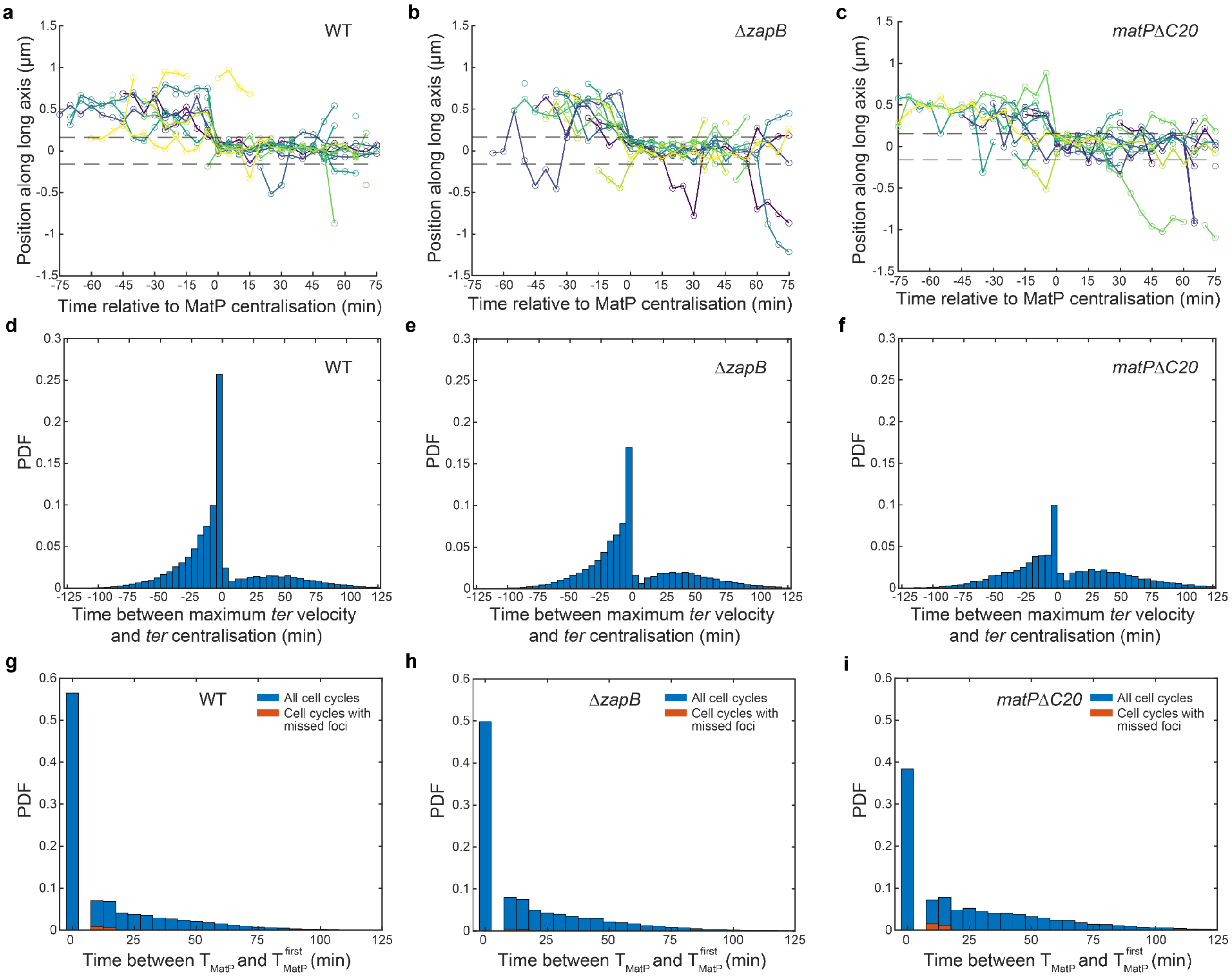

Supplementary Figure 5. **Analysis of ter centralisation. a** MatP foci tracks of ten randomly selected cell cycles belonging to wild-type strain (IS 130). **b** MatP foci tracks of ten randomly selected cell cycles from a Δ*zapB* strain. **c** MatPΔC20 foci tracks of ten randomly selected cell cycles from a *matPΔC20* strain. The dashed lines in **a**, **b** and **c** indicate the middle 4.8 pixels (~320 nm) used to define MatP centralisation **d** Distribution of time between the frame that has maximum MatP focus velocity (step-wise) towards mid-cell and the frame in which MatP centralization occurs for cell cycles belonging to wild-type strain. **e** Same as **d** but for Δ*zapB* strain. **f** Same as **d** but for *matPΔC20* strain. Negative values indicate the maximum MatP velocity occurred before the frame in which MatP centralisation occurs. Bin width is 5 minutes. **g** Distribution of the time between MatP centralisation and the time at which a MatP focus is detected in the middle 4.8 pixels for the first time for wild-type strain. **h** Same as **g** but for Δ*zapB* strain. **i** Same as **g** but for *matPΔC20* strain. The red coloured bars indicate the fraction of cell cycles in which the disparity can be explained by missed foci (between the two time points) causing the assignment of a later time of stable centralisation. Data

for (**a,d,g**), **(b,e,h**) and (**c,f,i**) are as in Figures 1 and Supplementary Figures 6 and 7 respectively. Source data are provided as a source data file.

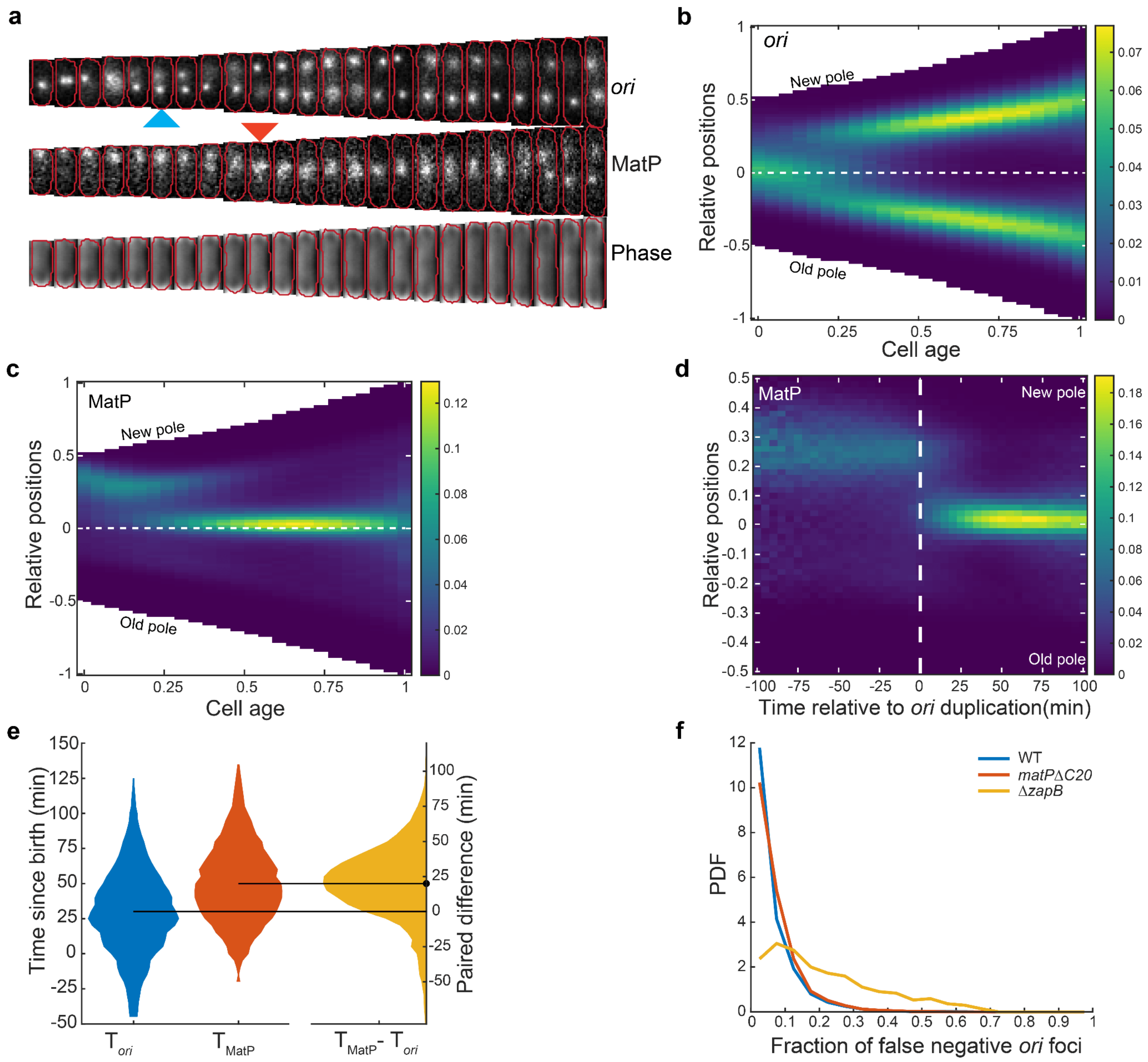

Supplementary Figure 6. **Analysis of *ΔzapB* strain.** **a** An example cell cycle of the *ΔzapB* strain (IS 161) with *ori* and MatP labelled. The blue arrow indicates *ori* focus duplication and the red arrow indicates MatP relocalization to mid-cell. **b** Average foci position kymograph of *ori* in the *ΔzapB* strain **c** Average foci position kymograph of MatP in the *ΔzapB* strain. **d** Kymograph of MatP foci positions synchronised to *ori* focus duplication. **e** Distribution of the time of *ori* focus duplication, T_{ori} (mean ± sd = 30.2 ± 30.8 min) and MatP centralization, T_{MatP} (50.6 ± 28.0 min) along with the time difference between the two events (20.4 ± 27.3 min) similar to Figure 1c. The horizontal lines indicate the median values of 30 and 50 minutes for T_{ori} and T_{MatP} respectively. The dot indicates the median (20 minutes) of the paired difference T_{MatP} - T_{ori}. **f** The distribution of false negative (missed foci) rate of *ori* foci in individual cell cycles for the indicated strains. The true number is based on the tracking inferred by *Track [1] (see methods). For unknown reasons the *ΔzapB* strain has more missed foci. This results in a later identification of *ori* duplication in some cells. To correct for this **d** and **e** use only cell cycles with a false negative fraction of less than 0.2 (n=19356 cell cycles). Otherwise, data as in Figure 5. The colour scale in **b, c** and **d** is as in Figure 1. Source data are provided as a source data file.

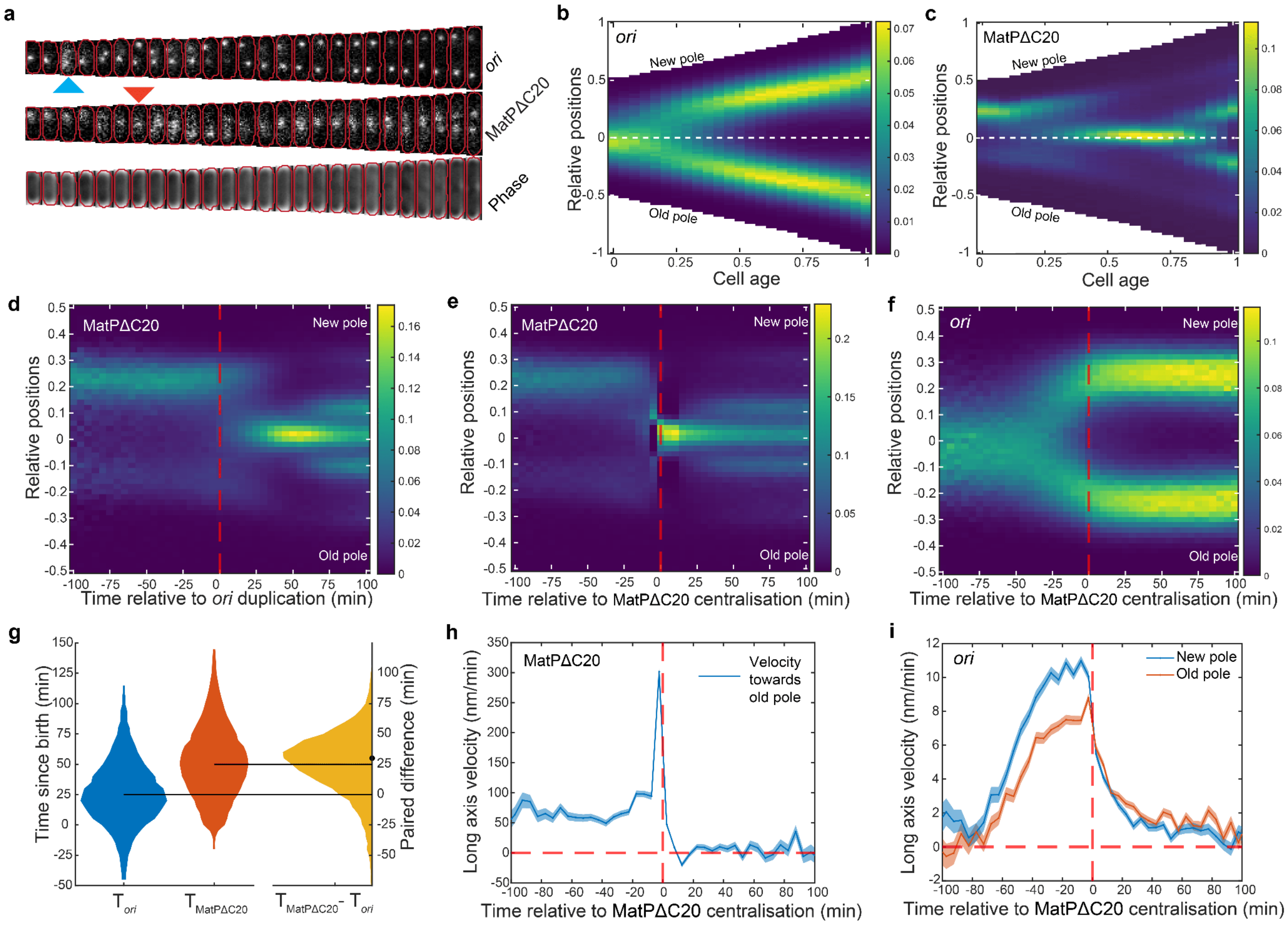

Supplementary Figure 7. **Analysis of *matPΔC20* strain. a** An example cell cycle of the *matPΔC20* strain (IS 146) with *ori* and MatPΔC20 labelled. The blue arrow indicates *ori* focus duplication and the red arrow indicates MatPΔC20 relocalization to mid-cell. **b** Average foci position kymograph of *ori* in a *matPΔC20* strain **c** Average foci position kymograph of MatPΔC20 in a *matPΔC20* strain. **d** Kymograph of MatPΔC20 foci positions synchronised to *ori* focus duplication. **e** Kymograph of MatPΔC20 foci positions synchronised to MatPΔC20 centralization. **f** Kymograph of *ori* foci positions relative to MatPΔC20 centralization. **g** Distribution of the time of *ori* focus duplication, T_{ori} (mean ± sd = 26.7 ± 26.8 min) and MatPΔC20 centralization, $T_{MatP\Delta C20}$ (52.4 ± 30.0 min) along with the time difference between the two events (25.8 ± 27.0 min) similar to Figure 1c. The horizontal lines indicate the median values of 25 and 50 minutes for T_{ori} and $T_{MatP\Delta C20}$ respectively. The dot indicates the median (30 minutes) of the paired difference $T_{MatP\Delta C20}$ - T_{ori}. **h** Mean velocity of MatPΔC20 focus track towards the old pole relative to MatPΔC20 centralisation. **i** Mean velocity of *ori* foci tracks towards the nearest pole relative to the time of MatPΔC20 centralisation. Data is from 24939 cell cycles. The colour scale in **b, c, d, e** and **f** is as in Figure 1. Source data are provided as a source data file.

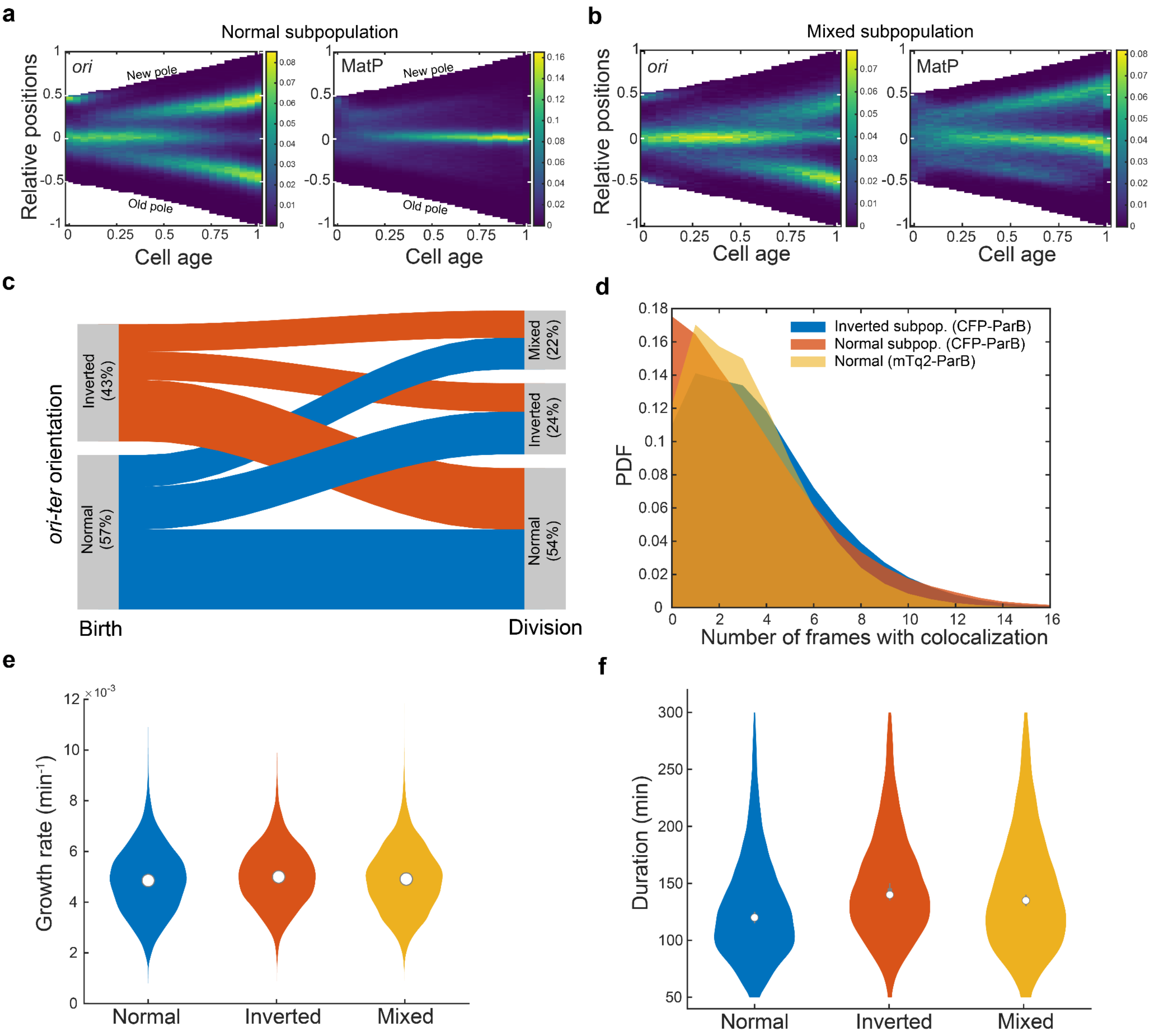

Supplementary Figure 8. **Analysis of CFP-ParB labelled cells.** **a** Foci positions kymographs of cells in the normal subpopulation (n=8269 cells) of CFP-ParB labelled cells (IS 111). **b** Foci position kymographs of cells in mixed subpopulation (n=3407 cells). The cells are oriented according to the position of the pole proximal MatP focus on the last frame of the cell cycle. **c** Flow diagram showing how frequently cells born with normal or inverted *ori-ter* orientation produce daughters with normal, inverted or mixed orientations (n=12384 cells). **d** Total number of frames in each cell cycle where MatP and *ori* foci are colocalized for the data shown in Figure 6d. The colocalization is defined as frames in which any MatP and *ori* foci appear within 260 nm. **e** The distributions of growth rates of the normal (mean ± sd = (4.9±1.3) x 10^{-3} min^{-1}), inverted ((5.0±1.2) x 10^{-3} min^{-1}) and mixed ((4.9±1.3) x 10^{-3} min^{-1}) subpopulations. The white circle indicates the mean. Growth rate is calculated using an exponential fit to the cell area for each cell cycle. **f** The distributions of cell cycle durations in normal (129 ± 46 min), inverted (148 ±46 min) and mixed (142 ± 50 min) subpopulations. The white circles indicate the mean. The normal, inverted and mixed subpopulations had 8269, 3774 and 3407 cell cycles respectively. Cell cycles shorter than 50 minutes and longer than 300 minutes were excluded from analysis (see methods). The colour scale in **a** and **b** is as in Figure 1. See also Figure 6. Source data are provided as a source data file.

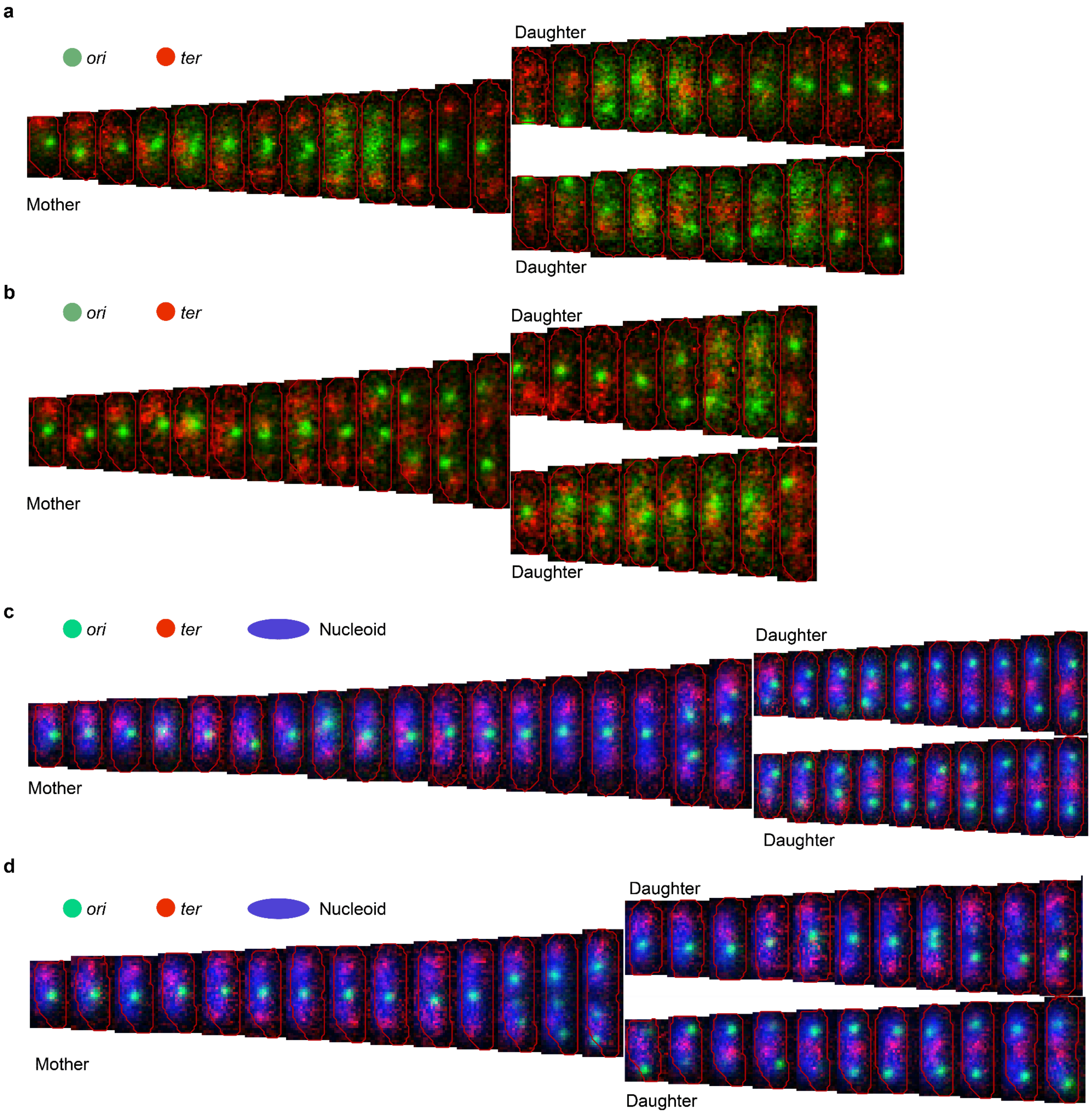

Supplementary Figure 9. **Cells with an inverted organisation segregate their chromosomes successfully. a** Representative cell cycle dividing with an inverted chromosome orientation (*ter* near poles rather than at middle towards division) along with its daughters which inherited an inverted chromosome orientation at birth (*ter* near old pole rather than at new pole) **b** Representative cell cycle dividing with a mixed chromosome orientation (*ter* near pole and mid-cell towards division) along with its daughters which inherited a normal (top) and an inverted (bottom) chromosome orientation at birth. Each frame is spaced by a 10-minute interval. Note that cell cycles for daughters may not constitute complete cell cycles. Data for **a, b** is from Figure 6. **c, d** Two representative examples of cell cycles with an inverted chromosome orientation in strain (IS 129) which has *ori*, *ter* and nucleoid labelled. The dataset is the same as in Figure 2. Each frame is spaced by a 10-minute interval. Despite the abnormal chromosome organisation, nucleoid constriction is observed prior to cell division in both mother cells. Following division, daughter cells grow normally. Anucleate cell production is comparable for cells born with normal (~0.25%) or inverted (~0.5%) orientations. Note that one daughter of the cell in **a, d** has an inverted orientation.

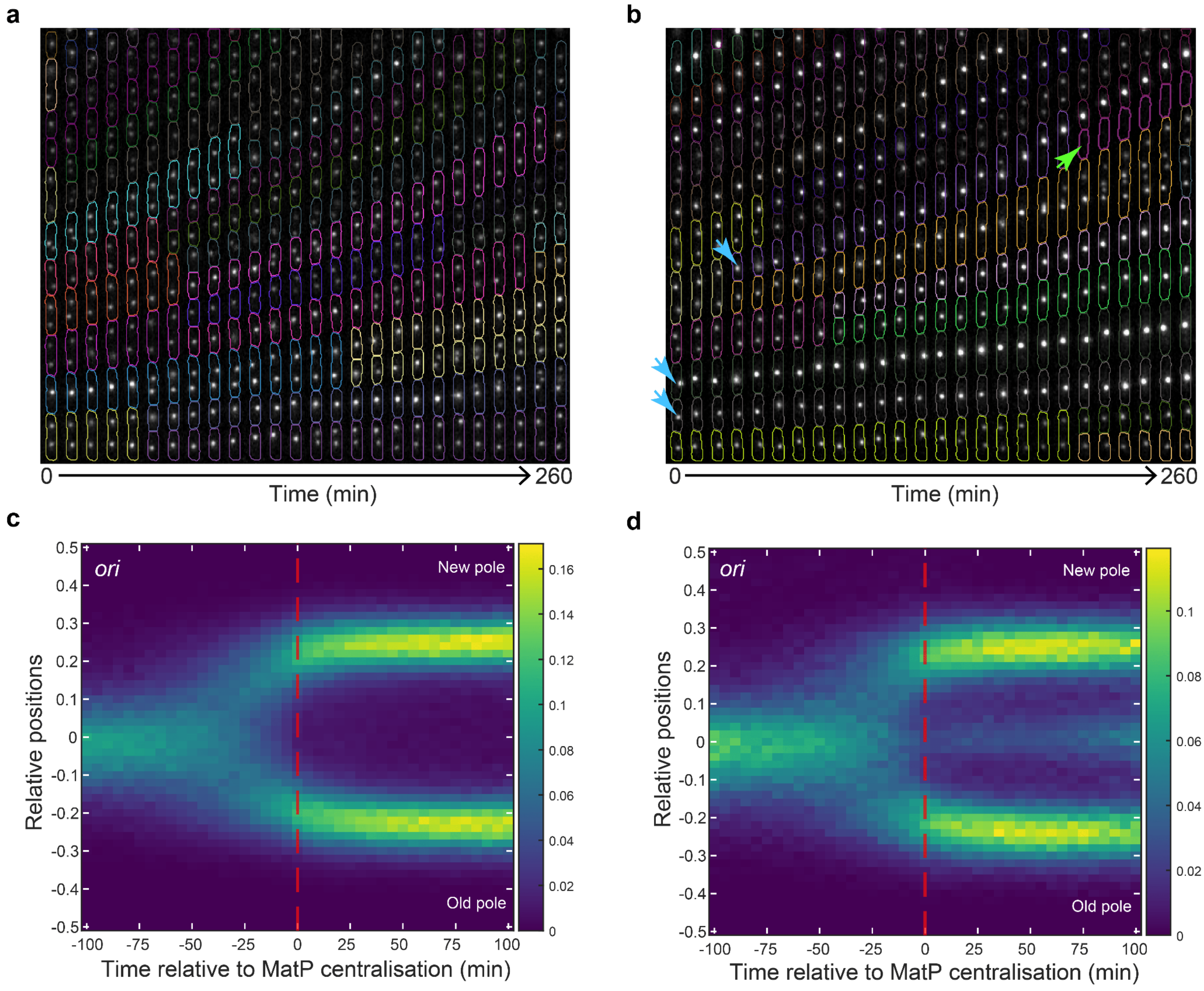

Supplementary Figure 10. **The effect of mild TopoIV overexpression. a** *ori* signal in a growth channel for wild-type strain (IS 130) grown in M9 glycerol+0.0005% arabinose. Use of glycerol instead of glucose is to avoid catabolite repression of the P_{BAD} promoter. Cells belonging to the same cell cycle are represented in contours of the same colour. Time interval between each frame is 10 minutes. Total duration shown is 260 minutes. **b** same as **a** but for a strain with additional arabinose-induced TopoIV expression (IS 252). Note that a greater fraction of cells in **b** have a single *ori* focus compared to **a.** Blue arrows indicate example cells that have a persistent (> 150 minutes) single *ori* focus. One such cell division resulting in the formation of an anucleate cell is marked with a green arrow. **c** Kymograph of *ori* foci positions relative to MatP centralization for IS 130 strain. **d** Kymograph of *ori* foci positions relative to MatP centralisation for IS 252 strain. Data is from n=8798 and 7175 cell cycles for strains IS 130 and IS 252 respectively.

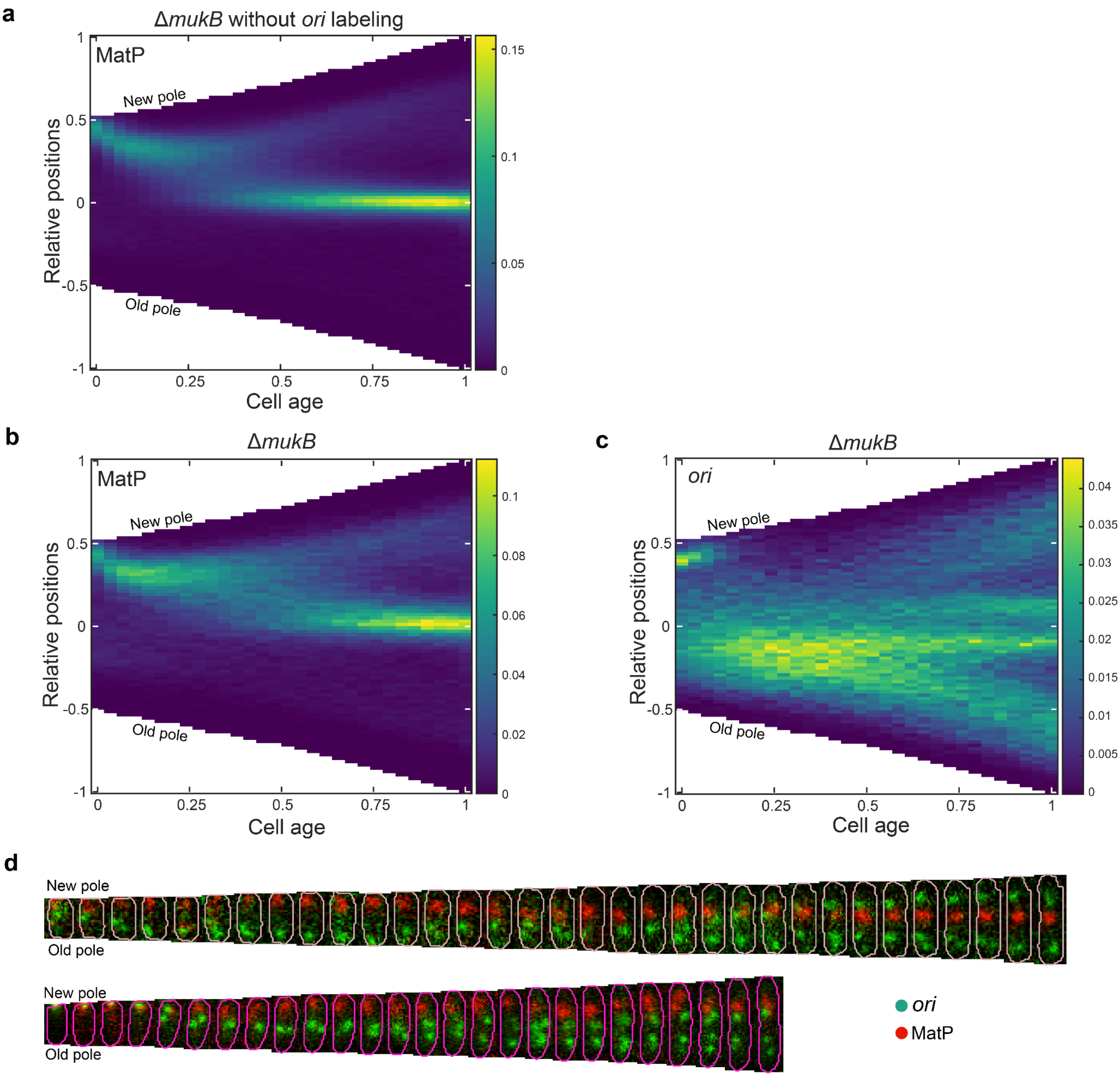

Supplementary Figure 11. **MukB deletion is not compatible with *ori* labelling by ParB. a** Foci position kymograph of MatP in a *ΔmukB* strain (IS 174) without the plasmid carrying mTurquoise2-ParB$_P$ used to label *ori* (n=11062 cell cycles). **b** and **c** Foci position kymograph of MatP (**b**) and *ori* (**c**) in a *ΔmukB* strain carrying the plasmid (n=3122 cell cycles). Cell cycles producing anucleate daughters (detected by the absence of MatP foci) are not included in the analysis. **d** Representative cell cycles showing MatP (red) and *ori* (green) dynamics in the *mukB* strain. The colour scale in **a, b** and **c** is as in Figure 1.

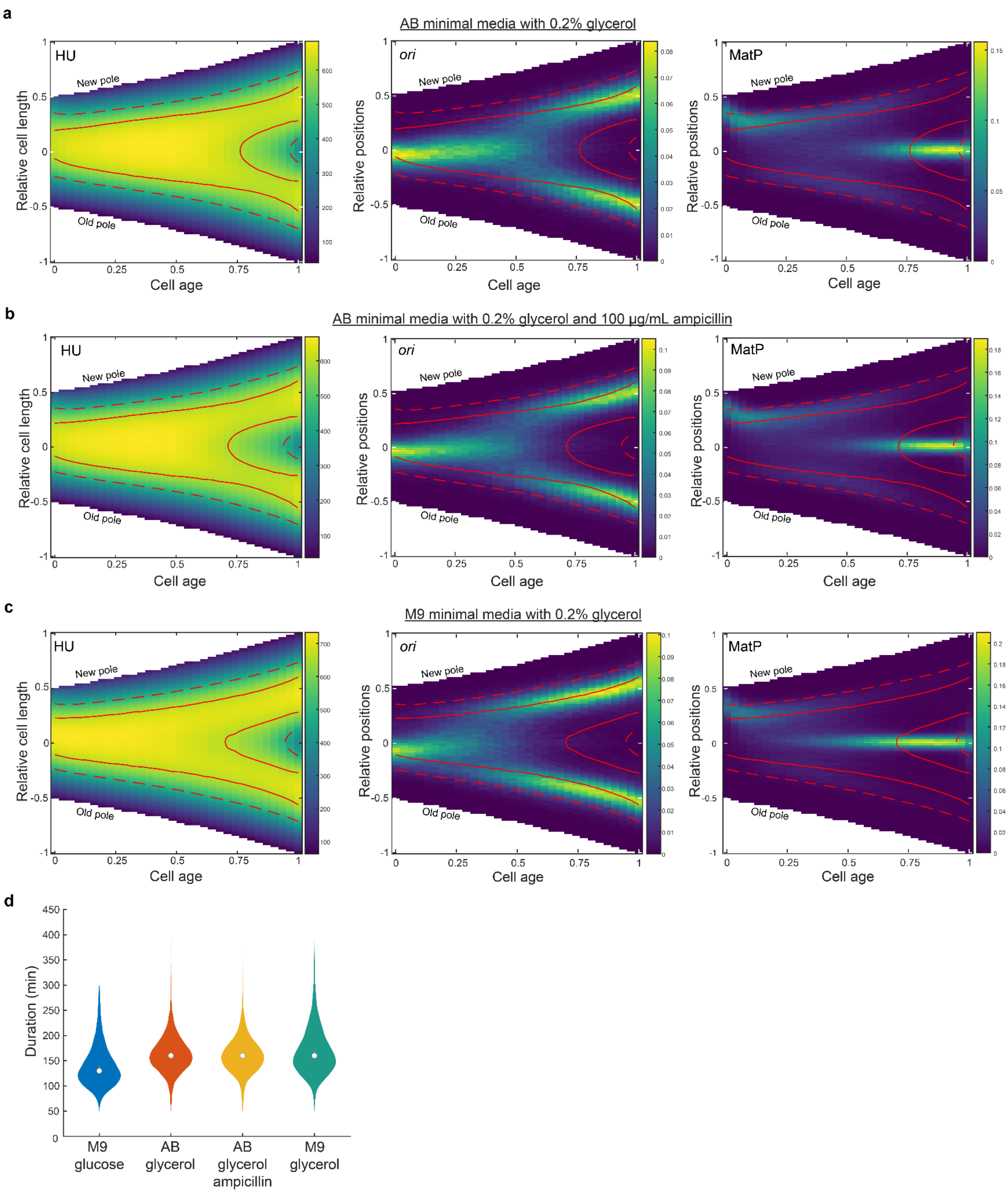

Supplementary Figure 12. **Asymmetry of ori positioning within the nucleoid is maintained during growth on glycerol. a** (Left) Average kymograph of HU-mCherry signal along the long axis of the cell. The solid contour lines represent upper 50 percent and the dashed lines represent upper 80 percent of the total HU-mCherry signal. Average kymograph of *ori* foci positions (middle) and MatP foci positions (right) with contour lines from HU-mCherry grown in AB minimal media with 0.2% glycerol, 1 µg/mL thiamine and 1 µg/mL uracil (n=7281 cell cycles). **b** Same as **a** but with the addition of 100 µg/mL ampicillin (n=8128). **c** Same as **a, b** but grown in M9 minimal media supplemented with 0.2% glycerol (n=6973). **d** Cell cycle duration of strain IS 129 in different media conditions. White dots indicate mean values: M9 minimal media with glucose (same as

Figure 2) (mean ± s.d = 140.7 ± 41.7 min), AB minimal media (162.8 ± 35.8 min), AB minimal media with ampicillin (161.1 ± 35.9 min) and M9 minimal media with 0.2% glycerol (169.6 ± 48.1 min). All experiments except M9 glucose were done at 32°C. The colour scales are as in Figure 2. Source data are provided as a source data file.

Supplementary Tables

Supplementary Table 1. Strains used in this study

Strain name	Genotype	Source
RM29	MG1655 *glmS::parS*$_{P1}$*::kan*	Ref. [2]
RH3	MG1655 *hupA-mCherry::frt matP-YPet::frt::kan::frt*	Ref. [3]
IS 111	MG1655 *matP-YPet::frt glmS::parS*$_{P1}$*::kan* + pFHCP1-CFP	This study
IS 129	MG1655 *hupA-mCherry::frt matP-YPet::frt glmS::parS*$_{P1}$*::kan* + pFHCP1-mTurquoise2	This study; MG1655 *hupA-mCherry::frt matP-YPet::frt::kan::frt* received from Jaan Männik[3]
IS 130	MG1655 *matP-YPet::frt glmS::parS*$_{P1}$*::kan* + pFHCP1-mTurquoise2	This study
IS 146	MG1655 *matPΔC20-YPet::frt glmS::parS*$_{P1}$*::kan* + pFHCP1-mTurquoise2	This study
IS 161	MG1655 *ΔzapB::frt::cat::frt matP-YPet::frt glmS::parS*$_{P1}$*::kan* + pFHCP1-mTurquoise2	This study
IS 173	MG1655 *ΔmukB::frt::kan::frt matP-YPet::frt glmS::parS*$_{P1}$*::frt::cat::frt*	This study
IS 174	MG1655 *ΔmukB::frt::kan::frt matP-YPet::frt glmS::parS*$_{P1}$*::frt::cat::frt* + pFHCP1-mTurquoise2	This study
IS 233	MG1655 *rhlE::parS*$_{P1}$*::frt elaD::parS*$_{pMT1}$*::frt::cat::frt* +pFHCP1-mTurquoise2-T1-mVenus	This study
IS 234	MG1655 *glmS::parS*$_{P1}$*::kan rhlE::parS*$_{pMT1}$*::frt::cat::frt* +pFHCP1-mTurquoise2-T1-mVenus	This study
IS 248	MG1655 *glmS::parS*$_{P1}$*::kan elaD::parS*$_{pMT1}$*::frt::cat::frt* +pFHCP1-mTurquoise2-T1-mVenus	This study
IS 252	MG1655 *matP-YPet::frt glmS::parS*$_{P1}$*::kan* + pFHCP1-mTurquoise2-TopoIV	This study

Supplementary Table 2. Plasmids used in this study

Plasmid name	Genotype	Use	Source
pFHCP1-CFP	pBR322 origin.<br>Relevant genes: *lacI*$_q$,<br>*lacO-cfp-parB*$_{P1}$.<br>IPTG inducible.<br>Ampicillin resistance.	Used to express CFP-ParB$_{P1}$ needed to visualize *ori*.	This study; Derived from plasmid pFHC2973[4]
pFHCP1-mTurquoise2	pBR322 origin.<br>Relevant genes: *lacI*$_q$,<br>*lacO-mTurquoise2-parB*$_{P1}$.<br>IPTG inducible.<br>Ampicillin resistance.	Used to express mTurquoise2-ParB$_{P1}$ needed to visualize *ori*.	This study; Made by replacement of *cfp* with mTurquoise2 in pFHCP1-CFP
pFHCP1-mTurquoise2-T1-mVenus	pBR322 origin.<br>Relevant genes: *lacI*$_q$,<br>*lacO-mTurquoise2-parB*$_{P1}$-*mVenus-parB*$_{pMT1}$.<br>IPTG inducible.<br>Ampicillin resistance.	Used to express mTurquoise2-ParB$_{P1}$ and mVenus-ParB$_{pMT1}$ needed to visualize *parS*$_{P1}$ and *parS*$_{pMT1}$ tagged chromosomal loci respectively .	This study; Made by replacement of *cfp* with *mTurquoise2* and *ygfp* with *mVenus* in pFHC2973 plasmid
pGBKD3-parSP1	pSC101 origin.<br>Relevant genes:<br>*parS*$_{P1}$::*frt*::*cat*::*frt*	Used as a template to clone *parS*$_{P1}$ sequence for chromosomal integration	Ref. [5]
pGBKD3-parSpmT1	pSC101 origin.<br>Relevant genes:<br>*parS*$_{pMT1}$::*frt*::*cat*::*frt*	Used as a template to clone *parS*$_{pMT1}$ sequence for chromosomal integration	Ref. [2]
pWX35	pBR322 origin.<br>Relevant genes: *araC*,<br>P_{BAD}, *parE, parC*<br>Ampicillin resistance	Template plasmid to amplify *araC*::P_{BAD}::*parE::parC* region to clone into pFHCP1-mTurquoise2 plasmid	Ref. [6]
pFHCP1-mTurquoise2-TopoIV	pBR322 origin.<br>Relevant genes: *lacI*$_q$,<br>*lacO-mTurquoise2-parB*$_{P1}$,<br>*araC, P_{BAD}, parC, parE* .<br>IPTG inducible<br>*mTurquoise2-parB*$_{P1}$.<br>Arabinose inducible *parC, parE* genes (TopoIV).<br>Ampicillin resistance.	Express mTurquoise2-ParB$_{P1}$ needed to visualize *ori*. Additionally contains TopoIV (*parC-parE*) under P_{BAD} promoter	This study; Made by cloning the TopoIV genes from pWX35 plasmid

Supplementary Table 3. Primers used in this study

Primer name	Sequence	Notes
IS_oligo_55	TTATAAGCTTatgcgagagtagggaactgccagg	pFH2973 forward for creating pFHCP1-CFP
IS_oligo_59	TTATAAGCTTTCTAGAGGATCCCCGGGTTAATAG	pFH2973 reverse for creating pFHCP1-CFP
IS_oligo_155	GAATTCGAGCTCAGTGTCGAGCAG	pFHCP1-CFP forward from parB linker for creation of pFHCP1-mTurquoise2
IS_oligo_156	GGTCTGTTTCCTGTGTGAAATTGTTAT	pFHCP1-CFP reverse upstream CFP for creation of pFHCP1-mTurquoise2
IS_oligo_157	CGGATAACAATTTCACACAGGAAACAGACCatgg tttctaaaggtgaagaactgtttacc	mTurquoise2 forward with pFHCP1-CFP overhang
IS_oligo_158	GAATACCTGCTCGACACTGAGCTCGAATTCtttgt acagttcatccatacctaaggtaat	mTurquoise2 reverse with pFHCP1-CFP overhang
IS_oligo_85	ctcaacgatgaaaccgaagagaatcagccagatagcggagagg aagaaTAgtcttgagcgattgtgtaggctgga	*mukB* knockout forward from pKD4
IS_oligo_86	gaaacggagttttcggaaaaagaaaaggcggcattgctgccgcctt aaTTcatatgaatatcctccttagttcctattcc	*mukB* knockout reverse from pKD4
IS_oligo_89	aatcgggacgaggatttttatccatcaacgccttgcaattcaggaga gGTgtcttgagcgattgtgtaggctgga	*zapB* knockout forward from pKD3
IS_oligo_90	ttacctgttggcctacacagtaaagaaattacgcggaagatgaagc gtAAcatatgaatatcctccttagttcctattcc	*zapB* knockout reverse from pKD3
IS_oligo_204	aagcacttgtcagcaatttgagaacacGGGgattgtgtaggctgg agctgc	Forward for amplifying *parS* from pGBKD3-parSP1 or pGBKD3-parSpmT1 for integration at *rhlE* locus
IS_oligo_205	tctcggtggcttatgggcggtaaaacaGATtatcccgtgacaggtc attcagac	Reverse for amplifying *parS* from pGBKD3-parSP1 or pGBKD3-parSpmT1 for integration at *rhlE* locus
IS_oligo_206	tcattgttaattaattcgaccagtcagCAAgattgtgtaggctggagc tgc	Forward for amplifying *parS* from pGBKD3-parSP1 or pGBKD3-parSpmT1 for integration at *elaD* locus
IS_oligo_207	actgtttagcaaactatgttcgaccagTCAtatcccgtgacaggtca ttcagac	Forward for amplifying *parS* from pGBKD3-parSP1 or pGBKD3-parSpmT1 for integration at *elaD* locus
IS_oligo_216	CCCGGCATCCGCTTACAGACAAGCTGTGACcgt caattgtctgattcgttaccaa	araC upstream forward from pWX35 plasmid with overhang for cloning into pFHCP1-mTurquoise2 plasmid
IS_oligo_217	AACCTCTGACACATGCAGCTCCCGGAGACGctct catccgccaaaacagcc	parC downstream reverse from pWX35 plasmid with overhang for cloning into pFHCP1-mTurquoise2 plasmid
IS_oligo_218	CGTCTCCGGGAGCTGCATGTGT	Forward for amplifying pFHCP1-mTq2 backbone to clone TopoIV from pWX35 plasmid

IS_oligo_219	GTCACAGCTTGTCTGTAAGCGGATGC	Forward for amplifying pFHCP1-mTq2 backbone to clone TopoIV from pWX35 plasmid

Supplementary References

1. Köhler, R., Sadhir, I. & Murray, S. M. ★Track: Inferred counting and tracking of replicating DNA loci. *Biophys. J.* **122**, 1–9 (2023).
2. Mercier, R. *et al.* The MatP/matS site-specific system organizes the terminus region of the E. coli chromosome into a macrodomain. *Cell* **135**, 475–85 (2008).
3. Männik, J., Castillo, D. E., Yang, D., Siopsis, G. & Männik, J. The role of MatP, ZapA and ZapB in chromosomal organization and dynamics in *Escherichia coli*. *Nucleic Acids Res.* **44**, gkv1484 (2016).
4. Nielsen, H. J., Ottesen, J. R., Youngren, B., Austin, S. J. & Hansen, F. G. The *Escherichia coli* chromosome is organized with the left and right chromosome arms in separate cell halves. *Mol. Microbiol.* **62**, 331–338 (2006).
5. Espeli, O., Mercier, R. & Boccard, F. DNA dynamics vary according to macrodomain topography in the E. coli chromosome. *Mol. Microbiol.* **68**, 1418–1427 (2008).
6. Wang, X., Reyes-Lamothe, R. & Sherratt, D. J. Modulation of Escherichia coli sister chromosome cohesion by topoisomerase IV. *Genes Dev.* **22**, 2426–2433 (2008).
